# Flower Extracts as Multifunctional Dyes in the Cosmetics Industry

**DOI:** 10.3390/molecules27030922

**Published:** 2022-01-29

**Authors:** Tomasz Bujak, Martyna Zagórska-Dziok, Aleksandra Ziemlewska, Zofia Nizioł-Łukaszewska, Kamila Lal, Tomasz Wasilewski, Zofia Hordyjewicz-Baran

**Affiliations:** 1 Department of Technology of Cosmetic and Pharmaceutical Products, Medical College, University of Information Technology and Management in Rzeszow, Sucharskiego 2, 35-225 Rzeszow, Poland; mzagorska@wsiz.edu.pl (M.Z.-D.); aziemlewska@wsiz.edu.pl (A.Z.); zniziol@wsiz.edu.pl (Z.N.-Ł.); klal@wsiz.edu.pl (K.L.); 2 Department of Industrial Chemistry, University of Technology and Humanities in Radom, Chrobrego 27, 26-600 Radom, Poland; tomasz.wasilewski@uthrad.pl; 3 Research and Development Department, ONLYBIO.life S.A., Wojska Polskiego 65, 85-825 Bydgoszcz, Poland; 4 ŁUKASIEWICZ Research Network—Institute of Heavy Organic Synthesis “Blachownia”, Energetykow 9, 47-225 Kedzierzyn-Kozle, Poland; zofia.hordyjewicz@icso.lukasiewicz.gov.pl

**Keywords:** plant extracts, natural dyes, cosmetics, biologically active dyes, anti-inflammatory properties, antioxidants

## Abstract

Flowers are a natural source of bioactive compounds that not only have antioxidant, anti-inflammatory, and anti-aging properties, but can also be used as natural dyes. For this reason, nowadays plants are widely used to produce natural cosmetics and foods. In these studies, the properties of the water extracts of *Papaver rhoeas* L., *Punica granatum* L., *Clitoria ternatea* L., *Carthamus tinctorius* L., and *Gomphrena globosa* L., as bioactive, natural dyes, were investigated. Plant flower extracts were tested for their antioxidant (ABTS and DPPH radical methods) and anti-inflammatory effects by determining the ability to inhibit the activity of lipoxygenase and proteinase. The extracts were tested for their cytotoxic effect on skin cells, using Alamar Blue and Neutral Red tests. The ability to inhibit the activity of enzymes responsible for the destruction of elastin and collagen was also studied. Research has shown that extracts have no toxic effect on skin cells, are a rich source of antioxidants and show the ability to inhibit the activity of elastase and collagenase enzymes. *P. rhoeas* extract showed the strongest antioxidant properties with IC50 value of 24.8 ± 0.42 µg/mL and 47.5 ± 1.01 µg/mL in ABTS and DPPH tests, respectively. The tested plants are also characterized by an anti-inflammatory property, for which the ability to inhibit lipoxygenase at a level above 80% and proteinase at the level of about 55% was noted. Extracts from *P. rhoeas*, *C. ternatea,* and *C. tinctorius* show the strongest coloring ability and can permanently dye cosmetic products, without significant color changes during the storage of the product.

## 1. Introduction

In recent years, there has been a growing interest in natural and ecological products. It is especially noticeable in the cosmetics, food, and other fast moving consumer goods (FMCG) industries. Based on the observation of the current market trends, more and more consumers are looking for natural products, which, in their opinion, are safer in use and more effective. For that reason, producers are forced to look for natural replacements of synthetically derived substances in order to prepare products that meet the requirements of the consumers [1]. A lot of substances like emulsifiers, rheology modifiers, or surfactants were substituted by their natural equivalents, but many raw materials are still a big problem for producers [1]. One of the most problematic ingredients are dyes, for which there are not too many effective solutions in the nature. Most of the dyes used by the food or cosmetics industry are continuously produced by chemical synthesis due to their lower cost and higher stability compared to the natural coloring agents.

Unfortunately, synthetic dyes have several disadvantages, the most important of which is their irritating and sensitizing potential as well as their negative impact on the environment [2,3,4,5]. Naturally sourced ones are safer for human health and the environment, but they are not stable and may change color during product storage. They are also sensitive to changes in pH, UV radiation, and changes in temperature [6,7,8]. Looking for new dyes of natural origin, attention was paid to extracts obtained from plant flowers with a strong color. Plant dyes in the form of extracts may be more stable and more resistant to color changes due to the presence in their composition, in addition to coloring substances, of ingredients that are able to prevent their oxidation under the influence of external factors, such as UV radiation or the action of free radicals. These are naturally occurring substances, mainly from the group of antioxidants, which can prevent changes in plant color and maintain an intense color even when exposed to strong UV radiation [5,7,9,10]. In the case of isolating individual color substances from plants (like, for example, in the case of betalains extracted from beetroot), the resulting dye is devoid of these components, and in a lot of cases it is necessary to add synthetic antioxidants to the final product to prevent color changes [11].

Antioxidants are a group of chemical compounds that play an important role in defending against oxidative stress. Their main function is neutralization of oxygen free radicals, called reactive oxygen species (ROS), which are highly reactive side products of metabolism.

A large proportion of antioxidants are plant-derived substances, which include, for example, phenolic acids or flavonoids. Some plant dyes also show an antioxidant effect, which, due to their properties, could replace synthetic pigments currently used in cosmetics. An example of such compounds can be anthocyanins, which belong to the flavonoids. They are present in the leaves, fruits, flowers of many plants, for example berries (chokeberries, blackcurrants, blueberries, and others), grapes, red chicory, etc., and give them blue, red, and purple colors [12,13,14]. Moreover, anthocyanins exhibit anti-inflammatory, antioxidant, and hepatoprotective properties and support the proper functioning of the cardiovascular system [12,13,14]. The presence of anthocyanins has also been demonstrated in *Punica granatum* L., *Clitoria ternatea* L., and *Papaver rhoeas* L. [15,16,17]. Betacyanins, which are responsible for the red-violet color, are found in leaves, flowers, roots, plant fruits, and in mushroom caps. These dyes show anti-cancer, antioxidant, and anti-inflammatory properties. Betacyanins include gomphrenin I, gomphrenin II, and gomphrenin III, which are found in *Gomphrena globosa* L. [18,19]. Another coloring compound that has an antioxidant effect is carthamin. It gives the plant organs a red color and is present, among others, in *Carthamus tinctorius* L. [20,21].

The aim of these studies was to investigate the properties of water extracts from plants that are the source of plant pigments. In preliminary studies, extracts from colorful flowers of 20 different plants were obtained, the use of which in cosmetic products is not prohibited. Among them, five extracts characterized by the strongest color and stability during exposure to UV radiation, changes in the pH of the aqueous solution, and the action of oxidizing agents (hydrogen peroxide) were selected. The extracts with the most preferable properties selected for further research were extracts of *Papaver rhoeas* L. (PRE), *Punica granatum* L. *(PGE)*, *Clitoria ternatea* L. (KTE), *Carthamus tinctorius* L. (CTE), and *Gomphrena globosa* L. (GGE). Bioactive compounds were determined for the listed plant extracts, as well as the antioxidant and anti-inflammatory properties. The extracts were tested for cytotoxic activity on fibroblasts and keratinocytes. The ability to reduce transepidermal water loss (TEWL) and the ability to inhibit the activity of enzymes responsible for the destruction of elastin and collagen were also studied. Obtained extracts were applied in the model make-up remover in micellar liquid form as the bioactive and multifunctional dyes. 

## 2. Results and Discussion

### 2.1. Determination of Bioactive Compounds by HPLC-ESI–MS/MS

A chromatographic method was developed to deepen the chemical structures of the active compounds.

The main phenolic compounds were determined in the negative-ion mode on the basis of mass-to-charge ratio (*m*/*z*) of the detected precursor ions and confirmed by resulting product ions from MS^2^ fragmentation using HPLC-ESI-MS/MS. The compounds were identified based on the resulting product ions. The MS data, MS/MS fragmentation profiles, and molecular formula were compared with authentic standards or literature data [22,23]. Table 1 lists the active compounds identified in the water extracts using HPLC-ESI-MS/MS.

The extracted ion chromatograms obtained in negative-ion mode for investigated extracts in water are presented in a supplementary file. The obtained results of HPLC-ESI-MS/MS revealed the presence of polyphenols, of which phenolic acids and flavonoids were a well-represented group. The characterized flavonoids were quercetin and kaempferol derivatives, while phenolic acids were caffeic, quinic, gallic, and caffeoylquinic acids (CQA) with two isomers: 3- and 5-CQA. Several other flavonoid glycosides including kaempferol-3-*O*-rutinoside and kaempferol-3-*O*-glucoside were also identified in the sample extracts.

Quinic acid, gallic acid, caffeic acid, 3-CQA, 5-CQA, and quercetin were quantified based on the calibration curve generated using peak areas of analytical standards in multiple reaction monitoring (MRM) mode. The obtained results are presented in Table 2.

Based on the sum of the determined compounds (Table 2), it was found that the aqueous extract of PGE was the most abundant in determined bioactive compounds. Quinic acid was determined in the highest amount in aqueous extract of CTE, while the aqueous extract of PGE was characterized by the highest content of gallic acid. Caffeic acid was the most abundant compound determined in the KTE extract.

### 2.2. Determination of Antioxidant Properties

Analysis of the composition of the extracts showed the presence of flavonoids and phenolic compounds, such as quinic acid, gallic acid, quercetin, rutin, caffeic acid, and others. These substances are known for their antioxidant properties, which has been demonstrated in many studies. The antioxidant activity of the extracts was examined in the next part of this research.

The first study was carried out using the ABTS•+ radical. From the obtained results, the IC_50_ point was determined for each of the plant extracts, as shown in Table 3. The lowest IC_50_ value was shown for PGE extract (24.8 µg/mL), and it was about 5.4 times lower than the value obtained for GGE, which was the highest. Therefore, PGE showed the best antioxidant capacity. Moreover, PRE and KTE achieved low IC_50_ values (65.5 and 63.3 µg/mL respectively), which contributes to their good antioxidant effect.

The second method used to determine the antioxidant activity was DPPH and the results were also presented as IC_50_ points. In this case, the lowest score was also obtained for PGE and the highest for GGE (Table 4). This value was about 4.3 times lower. IC_50_ for PRE and KTE extracts was only about 1.8 times higher than PGE extract.

In the next part of the research, the ability of extracts to reduce the production of reactive oxygen species in cells was examined. When the levels of reactive oxygen species in cells exceed the number of antioxidants, it leads to oxidative stress. ROS can damage DNA, proteins, and lipids, which may contribute to the development of diseases and increase the aging process. In these studies, the effects on intracellular ROS production were investigated on fibroblasts and keratinocytes, using fluorogenic H_2_DCFDA dye.

**Figure 1:**
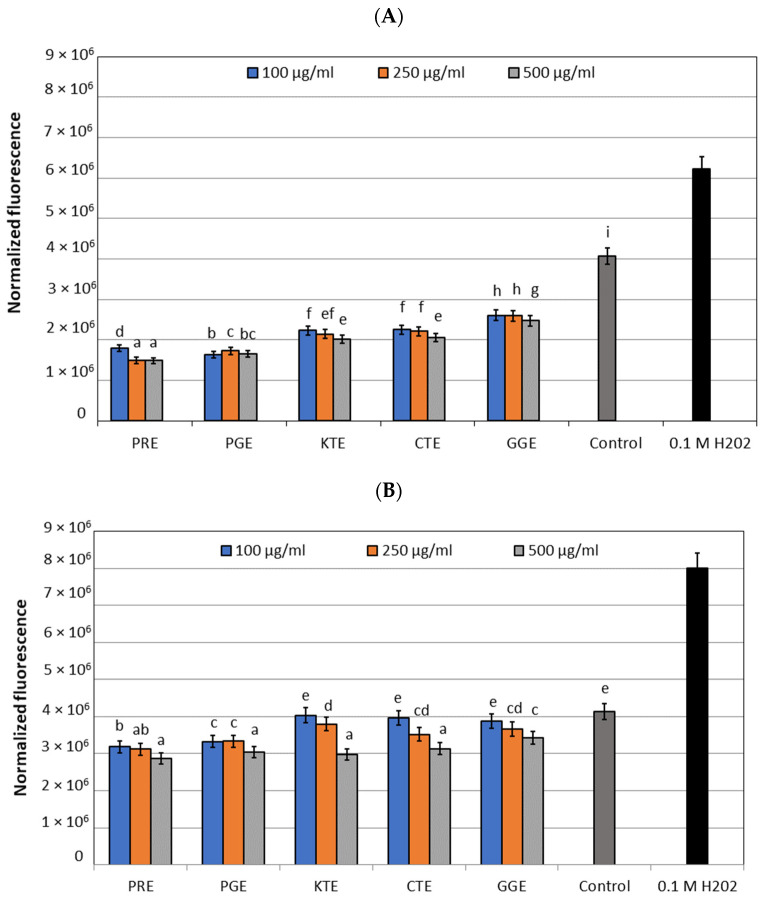
The effect of PRE, PGE, KTE, CTE, and GGE on the 20,70-dichlorofluorescein (DCF) fluorescencein BJ (**A**) and HaCaT cells (**B**). Different letters on the charts indicate significant differences between the individual results (*p* < 0.05).

By analyzing the results showed on the graphs (Figure 1A,B), it can be concluded that all tested extracts reduce the amount of ROS in cells. All extracts showed the highest potential to minimize the oxidative stress at the concentration of 500 µg/mL. In BJ cells, the strongest ability to reduce ROS was shown for PGE and PRE extracts. The fluorescence values for these plant extracts, with a concentration of 500 µg/mL, were around 60% lower than for the cells not treated with extracts (control). In HaCaT cells, the strongest ability to reduce ROS was also shown for PGE and PRE, and the fluorescence was 25–30% lower compared to the control (concentration of 500 µg/mL). The ability to reduce intracellular oxidative stress in HaCaT cells by KTE, CTE, and GGE at the concentration of 100 µg/mL was similar to the control.

Based on the described results, it can be confirmed that the tested plant extracts have an antioxidant capacity. This is due to the presence of various substances that are capable of neutralizing free radicals. The best antioxidative activity was shown by extracts of PGE and PRE. Water extract of *P. rhoeas* contained caffeic acid, quinic acid, gallic acid, rutin, and quercetin, which are known for their antioxidant properties [24,25,26,27,28,29]. Moreover, vitamin C has been shown to be present in the petals of this plant [30]. Vitamin C is an electron donor and by that it prevents oxidation of other compounds. As a result, it oxidizes itself, forming a relatively stable free radical. Due to these actions, it reduces oxidative damage [31,32]. PRE also contains pigments from the group of anthocyanins [33], which have the ability to scavenge free radicals [34]. The presence of the above-mentioned compounds in PRE gives this plant good antioxidant properties, which was demonstrated in these studies and by other researchers [35,36]. Analysis by HPLC-ESI-MS showed that the PGE water extract contained caffeic acid, quinic acid, quercetin, and kaempferol-*O*-glucoside. In addition, the flowers of these plants are rich in ellagic acid, ursolic acid, maslinic acid, and asiatic acid. These substances are known for their antioxidant capacity as well as anti-inflammatory properties [24,25,26,27,28,29,37,38]. It is their presence that makes the extract of this plant show its positive effect on reducing oxidative stress [39,40]. CTE contains caffeic acid, quinic acid, gallic acid, caffeoylquinic acids, isoquercetin, quercetin, rutin, and kaempferol-*O*-glucoside, also anthocyanins that are responsible for its antioxidant properties. Kamkaen and Wilkinson also proved the antioxidant activity of CTE using the DPPH method, obtaining the result for the water extract IC_50_ = 1 mg/mL [41]. GGE in addition to phenolic compounds and flavonoids determined using the HPLC-ESI-MS method, also contains betacyanins, which are pigments with antioxidant properties [18,42]. Susilaningrum and Wijayanti have shown that ethanol extract of GGE has very strong antioxidant activity (IC_50_ = 49.9 μg/mL) [43]. CTE contains caffeic acid, quinic acid, gallic acid, caffeoylquinic acids, isoquercetin, quercetin, kaempferol-*O*-glucoside, which make this plant exhibit antioxidant capacity.

### 2.3. Assessment of Matrix Metallopeptidase Inhibition

**Figure 2:**
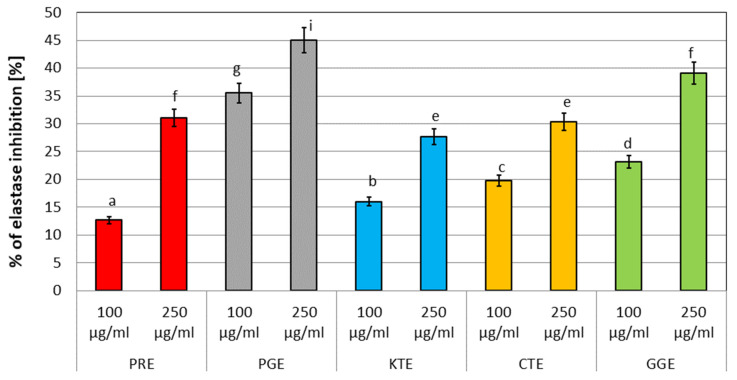
The effect of plant extracts on the activity of the elastase enzyme. Data are the mean of three independent experiments in which each sample was tested in triplicate. Different letters on the charts indicate significant differences between the individual results (*p* < 0.05).

**Figure 3:**
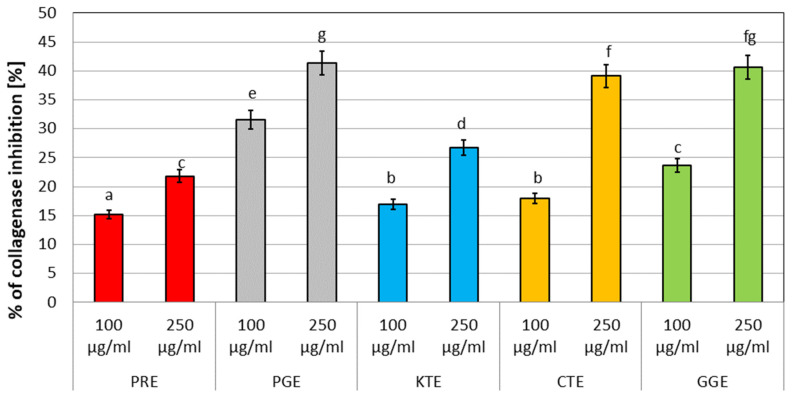
The effect of plant extracts on the activity of the collagenase enzyme. Data are the mean of three independent experiments in which each sample was tested in triplicate. Different letters on the charts indicate significant differences between the individual results (*p* < 0.05).

In order to assess the possibility of using plant extracts in formulations intended to combat the signs of skin aging, an important element is to assess their ability to inhibit the activity of enzymes closely involved in the skin aging processes. The main enzymes whose increased activity leads to the degradation of collagen and elastin fibers, which accelerates skin aging, are collagenase and elastase [44]. As part of this work, the influence of the analyzed extracts from five studied plants on the possibility of statistically significant inhibition of the activity of these metalloproteinases was investigated. As part of the conducted experiments, measurements were made for two concentrations of each of the extracts: 100 and 250 µg/mL and the results are presented in Figure 2 and Figure 3. It was observed that all of the analyzed extracts are able to a greater or lesser degree influence the activity of these enzymes in in vitro conditions. It was noted that at the higher of the tested concentrations, the anti-aging activity was greater. During the measurements of elastase activity, the greatest inhibition was observed for the PGE extract (44.97%), followed by GGE (39.11%), PRE (30.99%), CTE (30.33%), and KTE (27.7%), respectively. In the case of the second enzyme, collagenase, the PGE extract (41.30%) also showed the greatest inhibition, followed by GGE (40.61%), CTE (39.09%), KTE (26.68%), and PRE (21.83%). As part of the analyzes, measurements were also made for commonly known inhibitors of these enzymes, SPCK for elastase and 1,10-phenanthroline for collagenase, for which inhibition of 57.88% and 51.84% was observed, respectively. Thus, the inhibition obtained for the analyzed extracts, especially PGE and GGE, indicates that they exhibit only slightly lower activity than the commonly known inhibitors of these metalloproteinases, which may indicate their use in cosmetic and pharmaceutical preparations used against skin aging.

We have already demonstrated the anti-collagenase and anti-elastase activity of the studied plants in previous studies for a different type of extract (water-ethanol) [45]. The activity confirmed in this study also for aqueous extracts indicates that various types of extracts obtained from these plants can be a source of biologically active compounds with anti-aging activity. Chromatographic analyzes of the tested extracts showed the presence of numerous compounds with proven anti-aging properties, such as caffeic acid, quinic acid, gallic acid, quercetin, or rutin. The ability to inhibit skin aging is related to the wide spectrum of action of these compounds, which has been shown in numerous scientific papers. Chiang et al. in their study indicated that caffeic acid can inhibit skin photoaging as a result of UVB radiation by inhibiting metalloproteinases and increasing the production of I-type procollagen [46]. Moreover, Staniforth et al. showed that this phenolic acid can inhibit UVB-induced IL-10 mRNA expression and decrease mitogen-activated protein kinases activation [47]. The possibility of a slight inhibition of elastase activity by quinic acid was shown in the studies by Shoko et al. [48]. Chaikula et al. in their work demonstrated anti-aging properties of gallic acid manifested by inhibition of melanin formation by suppressing the activity of tyrosinase and tyrosinase-related protein-2, high antioxidant properties and the possibility of inhibiting matrix metalloproteinase-2 [49]. Moreover, Hwang et al. found that this acid reduces skin dryness and limits the formation of wrinkles. This is the result of inhibition of the secretion of matrix metalloproteinase-1 and an increase in the level of elastin, type I procollagen and transforming growth factor-β1 [50]. Other authors have shown that quercetin inhibits the activity of elastase and reduces lipid peroxidation [51,52]. The bioflavonoid rutin is also characterized by a very strong anti-aging effect. As shown by Seong et al., it can increase the expression of type I collagen mRNA and decrease the expression of matrix metallopeptidase 1 mRNA in human dermal fibroblasts. Moreover, rutin can positively affect skin elasticity and significantly reduce the number and length of wrinkles [53]. Thus, the possibility of the interaction of compounds presents in the extracts tested in this study on many cellular processes results in anti-aging properties of these plants. The ability of test plants to inhibit collagenase and elastase activity may involve several mechanisms. This may be related to the interaction of the polyphenolic compounds present in the extracts, mainly their hydroxyl groups, with the enzyme skeleton or side chains, or conformational changes that lead to the inactivation of the enzyme [54,55]. The inhibition may also be related to the ability of polyphenolic compounds and flavonoids to chelate metal ions that are found in the active site of metalloproteinases such as elastase and collagenase [56,57].

### 2.4. Determination of Anti-Inflammatory Properties

Over the past few decades, inflammation has been recognized as a major risk factor for various human diseases. Chronic inflammatory responses are predisposed to a pathological progression of chronic illnesses characterized by infiltration of inflammatory cells, excessive production of cytokines, dysregulation of cellular signaling, and loss of barrier function. Targeting reduction of chronic inflammation is a beneficial strategy to combat several human diseases. Proteinases and lipoxygenases are the enzymes that take part in various types of inflammation. Proteinases have been linked to arthritic reactions. Neutrophils, in their lysosomal granules, carry many serine proteinases. Leukocyte proteinases play a significant role in the development of tissue damage during inflammatory processes [58]. Lipoxygenases are key enzymes in the biosynthesis of leukotrienes, which in turn are crucial mediators in many inflammatory diseases. The mechanism of anti-inflammatory action involves a number of issues in which the metabolism of arachidonic and linoleic acids plays an important role [59,60]. 

**Figure 4:**
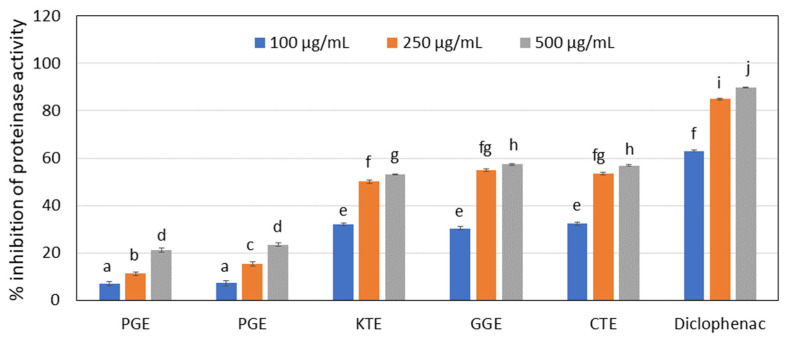
Proteinase inhibitory activity of tested plant extracts. Data are the mean of three independent experiments. Different letters on the charts indicate significant differences between the individual results (*p* < 0.05).

**Figure 5:**
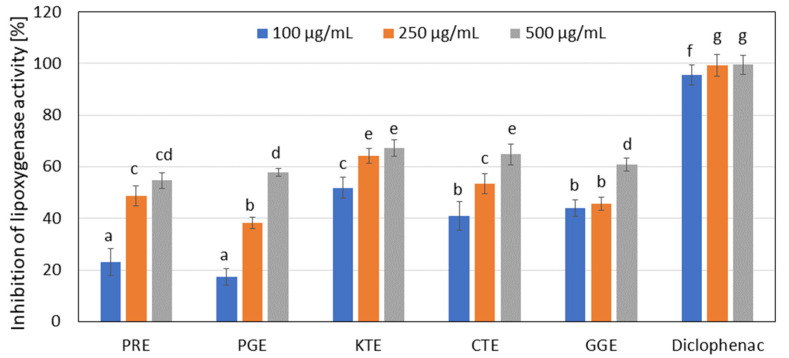
Lipoxygenase inhibitory activity of tested plant extracts. Data are the mean of three independent experiments. Different letters on the charts indicate significant differences between the individual results (*p* < 0.05).

Many natural products are used in traditional medical systems to treat the relief of symptoms from pain and inflammation, [61] therefore, the effect of the extracts analyzed on the inhibition of lipoxygenase and proteinase activity was investigated. In the range of concentrations analyzed (100–500 µg/mL), CTE water extract showed the strongest ability to inhibit proteinase (Figure 4) (at 57% for a concentration of 500 µg/mL). This activity was compared to the well-known proteinase inhibitor diclofenac, used as a control (about 89% inhibition at the highest concentration tested). However, similar results were obtained for GGE and KTE extract (about 56% and 53%, respectively, for the highest concentrations). Lower proteinase inhibition was observed for PRE and PGE extracts. In a further test measuring the ability to inhibit lipoxygenase, the aqueous extracts from KTE and CTE show the highest values (about 67% and 64% inhibition, respectively, for a concentration of 500 µg/mL). Diclofenac was also used as a control. GGE, PGE, and PRE extracts also feature significantly high values (60%, 57% and 54%, respectively). It was also noted that the ability to inhibit LOX and proteinase enzymes depends on the extract concentration (Figure 5).

Previous studies indicated that many polyphenolic compounds significantly contributed to the anti-inflammatory activities of many plant extracts [62]. Studies have shown the involvement of ROS in the inflammatory process, and phenolic compounds such as: gallic and quinic acid may block arachidonic acid metabolism by inhibiting the activity of lipoxygenase activity, or they may serve as scavenging reactive free radicals, which are produced during arachidonic acid [63]. The results obtained by BenSaad et al. indicate that ellagic acid, gallic acid, and punicalagin A&B isolated from *P. granatum* inhibited the production of nitric oxide (NO), prostaglandin E2 (PGE2), and interleukin 6 (IL-6) in lipopolysaccharide (LPS)-induced RAW 267.4 macrophages. Whether these compounds work as sole agents or have a synergistic effect still remains a question [64]. Since many flavonoids show anti-inflammatory properties, due to their intrinsic antioxidant behavior, they have been implicated in various inflammatory disorders. In particular, quercetin is the most interesting molecule because it interferes with specific biological pathways. Moreover, studies suggest that it can reduce the inflammatory process involved in several models through different mechanisms [65]. In particular, the AMP-activated protein kinase and the histone/protein deacetylase (AMPK/SIRT1) pathway results in more interesting inflammation management. Thus, AMPK activators can reduce macrophage inflammation. Quercetin and other flavonoids, as activators of AMPK and SIRT1, may reduce inflammation by interfering with this pathway [66]. It has been also shown that quercetin and quercetin monoglucosides exert the higher LOX inhibition potential [67]. Nair et al. have shown anti-inflammatory properties of KTE extract evaluating the presence of flavonols with the quercetin moiety such as: manghaslin Qu 3-[2G] rhamnosylrutinoside, Qu 3-*O*-dirhamnoside, and rutin. These molecules have shown strong inhibition of COX-2 activity and partial ROS suppression. In general, polyphenols present in CTE showed anti-inflammatory properties in LPS-induced inflammation in RAW 264.7 macrophage cells [68].

### 2.5. Cytotoxicity Assessment

**Figure 6:**
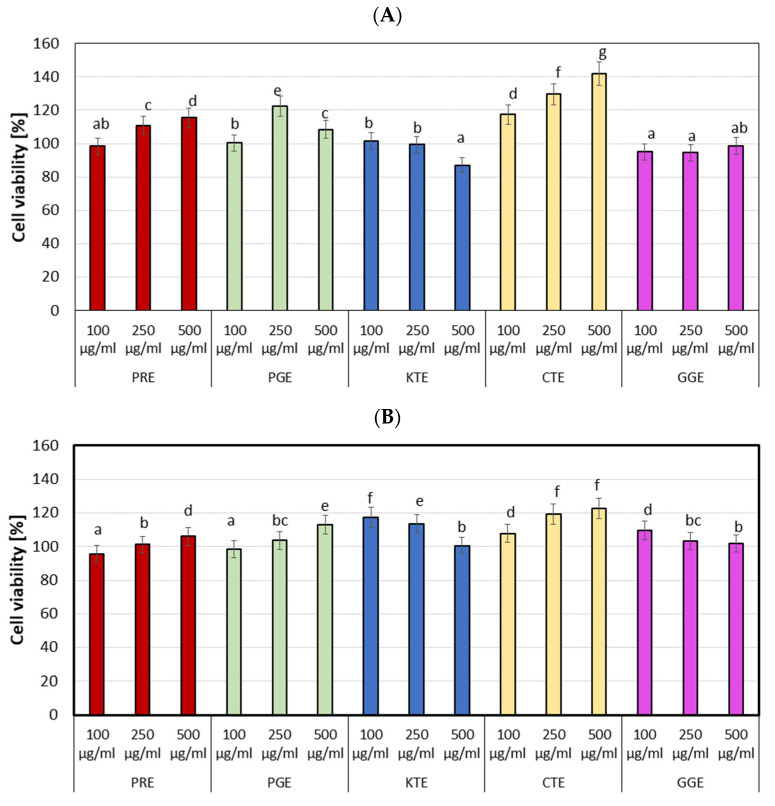
The effect of increasing concentrations of PRE, PGE, KTE, CTE, and GGE extracts on Neutral Red Dye uptake in cultured fibroblasts (**A**) and keratinocytes (**B**) after 24 h of exposure. Data are the mean ± SD of three independent experiments each consisting of four replicates per test group. Different letters on the charts indicate significant differences between the individual results (*p* < 0.05).

Creating new cosmetic raw materials, one of the most important properties is their safety of use. Substances dedicated for use in cosmetics must be non-toxic, especially in relation to skin cells, such as keratinocytes and fibroblasts. Two types of tests have been used to determine the toxicity of the analyzed extracts on HaCaT and BJ cells. The first study, using the neutral red uptake assay, enables to assess the viability of the cells treated with the analyzed extracts. This dye enters the lysosomes of a living cell and is released into the cytoplasm of dead cells. It was observed (Figure 6) that the CTE extract has the highest ability to increase proliferation of both the HaCaT and the BJ cells. In comparison with the control, this extract achieved about 20% and 40% higher values of the tested parameter at the concentration of 250 µL/mL (HaCaT and BJ) and 500 µL/mL (BJ cells), respectively. The GGE extract at concentration of 100 and 250 µL/mL and the KTE extract at the concentration of 500 µL/mL were characterized by little toxic effect on BJ cells. Other extracts had a positive influence on the viability of this cells. The PRE and PGE extracts at concentration of 100 µL/mL did not differ significantly from the control, and they increased the proliferation of BJ cells by about 10–15% compared to the control at the concentration of 250 and 500 µL/mL. In the case of keratinocytes, no decrease in cell viability was observed. For PGE, PRE, and CTE extracts, an increase in proliferation with increasing concentration was noted, while decrease in cell viability was demonstrated with increasing concentration of the KTE and GGE extracts.

**Figure 7:**
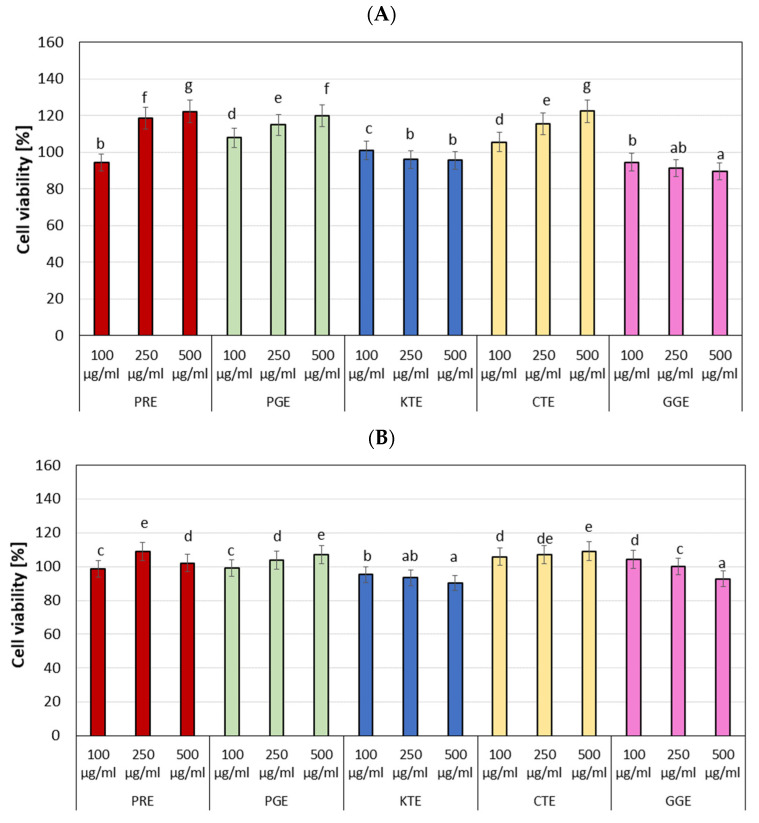
The reduction in resazurin after 24-h exposure to PRE, PGE, KTE, CTE, and GGE extracts in cultured fibroblasts (**A**) and keratinocytes (**B**). Data are the mean ± SD of three independent experiments each consisting of four replicates per test group. Different letters on the charts indicate significant differences between the individual results (*p* < 0.05).

The second test carried out to determine the cytotoxicity of the tested extracts was the resazurin test (Alamar Blue). It has been shown (Figure 7) that the viability of cells depends on the concentration of the extract with which the cells were incubated. In the case of fibroblasts, the PRE, PGE, and CTE extracts cause higher cells proliferation with an increase in their concentration. At the highest analyzed concentration (500 µL/mL), about 20% higher proliferation was observed comparing with the control. In the case of KTE and GGE extracts, a decrease in cell viability was observed with an increase in the concentration of extracts. These extracts showed little toxic effect on BJ at concentrations of 250 and 500 µL/mL. In case of keratinocytes, a similar effect of the analyzed extracts on the skin cells was observed, but their ability to proliferate was not as strong as in the case of fibroblasts. The highest ability to increase keratinocytes proliferation was observed for the CTE extract in the entire range of analyzed concentrations. Similar values were obtained for the PRE extract at a concentration of 250 µL/mL and for the PGE extract at a concentration of 500 µL/mL. The KTE extract showed significantly higher toxic effect on HaCaT cells than the GGE extract.

The analyzed extracts have not been extensively tested for their toxicity to skin cells before. There are only a few cytotoxicity studies on extracts or their main active ingredients, especially towards cancer cells. The authors of previous studies indicated that these extracts generally do not have a toxic effect on skin cells, and their ability to increase cell proliferation is most often attributed to the high content of polyphenols, anthocyanins, and flavonoids [45,69,70,71,72,73]. Some authors also indicated that the individual components contained in the extracts may exhibit a toxic effect on skin cells, while the extract as a whole does not. Ali Hijazi et al. [69] showed that alkaloids extracted from *Punica granatum* are toxic to normal and cancer cell lines, while the whole extract is less toxic. The study of Nasiri et al. [70] indicates that *Punica granatum* flower extract may be useful in accelerating the wound healing process due to its ability to increase the proliferation of skin cells. In our previous research [45], it was shown that the water-ethanolic extracts obtained from the analyzed plants were characterized by a higher ability to increase the proliferation of BJ cells, and the KTE and GGE extracts showed a lower toxic effect on these cells. The effect of water-ethanol extracts on HaCaT cells was similar to that of pure aqueous extracts, but in the case of extracts obtained with ethanol, slightly more favorable proliferation properties were observed than in the case of aqueous extracts. The differences between the composition of both types of analyzed extracts may cause differences in their toxicity. As shown, water extracts are not as rich in bioactive ingredients as water-ethanol extracts. The greatest differences are observed in the content of rutin and isoquercetin.

### 2.6. Determination of Sun Protection Factor

The unfavorable effects of UV radiation on the skin may be revealed both shortly after exposure as well as even years later. The solar radiation has an immunosuppressive effect, which accelerates the aging process of the skin with all the consequences associated with it, including increased carcinogenesis [74]. The currently observed trends indicate a growing need to develop products characterized not only by a very high level of safety in use, but also multifunctionality in the sense that products will feature the skin’s anti-radiation protection with broader scope of action than used so far. Some plant substances play an invaluable role in this aspect, which are able not only to provide sun protection, but also to neutralize the already existing negative effects of solar radiation on the skin [75,76,77]. The conducted research showed that the analyzed plant extracts PRE, PGE, KTE, CTE, and GGE are characterized by high SPF coefficients. 

The analysis of the coefficients (SPF) was carried out for the obtained water extracts from the above-mentioned plants at concentrations of 10 and 50 mg/mL. For each of investigated plants higher concentration of the extract resulted in significantly higher value of SPF. Comparison between extracts shows that the highest values of the SPF were observed for the KTE extract, which holds for both investigated concentrations. Another interesting observation is that even in the lower of investigated concentrations the KTE extract still exhibited high SPF, while the values for other extracts decreased noticeable. This is clear when we analyze how much the result for KTE was higher from other extracts. For the concentration of 50 mg/mL, the SPF was higher by the factor 1.2 (when compared to PGE, CTE) to the factor almost 1.9 (when compared to PRE, GGE). The same calculation for the concentration of 10 mg/mL will give factors from 1.4 (when compared to PGE) to factors of the range 2–3 (when compared to CTE, GGE), even up to factors like 10 when compared to PRE. When focusing on the KTE extract, one can notice that a decreasing concentration of the extract by factor 5 (from value 50 mg/mL to value of 10 mL/mL) results in andecrease of the SPF value by factor 3.4, which means that SPF decreases slower than the concentration.

**Figure 8:**
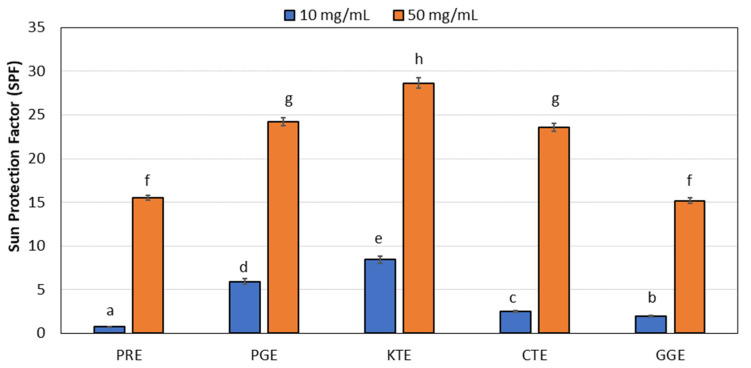
Ability of PRE, PGE, KTE, CTE, and GGE extracts to protect against UV radiation. Different letters on the charts indicate significant differences between the individual results (*p* < 0.05).

Above shows that the KTE extract can be efficient even when applied in low concentrations (Figure 8).

### 2.7. Transepidermal Water Loss (TEWL) and Skin Hydration Measurements

Due to the wide range of biological and pharmacological activity of plant raw materials, plant substances contained in extracts have a significant impact on the condition of the skin. In particular, we are talking here about the influence of secondary metabolites on the condition of our skin [78,79]. 

**Figure 9:**
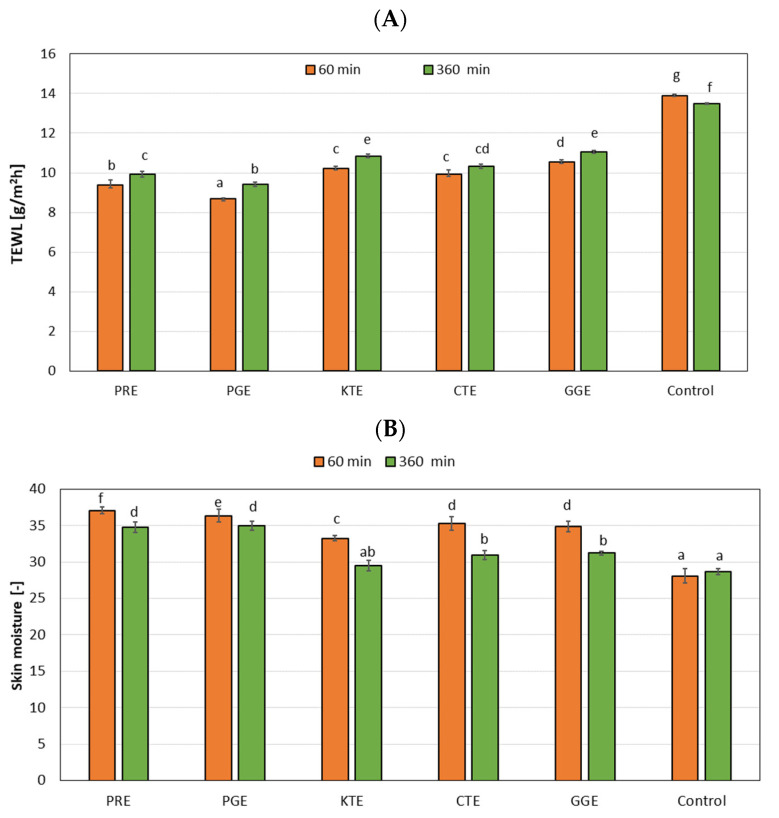
Influence of PRE, PGE, KTE, CTE, and GGE extracts on skin hydration (**A**) and TEWL (**B**). Different letters on the charts indicate significant differences between the individual results (*p* < 0.05).

In the next stage of the research, the analyses of hydration and TEWL were carried out. The effect of the tested extracts on the skin was assessed. Measurements were made at two-time intervals of 60 and 360 min for the extract concentration of 10 mg/mL. For the TEWL measurement, the greatest percentage decrease was shown for PGE extract, where control value of 13.9 fallen to 8.71, resulting in the percentage decrease of 37%. It is also worth to analyze the KTE extract from that perspective, as this was the one exhibiting the highest SPF values. For the KTE extract, the TEWL control value decreased to the value of 10.22, which means decrease of 26% (Figure 9A).

However, in the case of the second instrument measurement, it was shown that the analyzed extracts cause an increase in moisturization in relation to the control sample, both after 60 as well as after 360 min.

As a result of the analyses, it was found that the analyzed PRE, PGE, KTE, CTE, and GGE extracts increase skin hydration (Figure 9B). An increase in moisturizing properties was observed along with an increase in the concentration of the extract in the preparations. The strongest moisturizing properties have been observed for PRE and PGE extracts equaling 32% and 29% after 60 min, and equaling 21% and 22% after 360 min. The lowest level of skin hydration, though, has been found for KTE extract equaling 18% after 60 min and close to 3% after 360 min.

### 2.8. Application Analysis

#### 2.8.1. Determination of the Color Parameters of Extracts

Due to their natural color, the active ingredients of plant flowers can be used as natural dyes in many commercial products, such as cosmetics and food. Color analysis was performed for the obtained extracts (Table 5).

PRE, KTE, and CTE extracts were found to have the highest potential as natural cosmetic pigments. However, the highest chroma values (C*) was observed for CTE. Based on the value of the *h^o^* parameter, it was found that it is the yellow color of this extract that is observed and visible to the naked eye. In the case of KTE and PRE extracts, despite the low C* value (2.8), the color of these extracts can be clearly seen with the naked eye and was specified as red-purple for PRE and blue-violet for KTE extract. The water extracts of PGE and GGE were reddish and slightly orange in color, and the obtained chroma values were at the level of 1.3. For this color, these values were not significantly discernible to the naked eye.

#### 2.8.2. Determination of the Color Parameters of Cosmetics Based on the Extracts

In recent years, there has been a strong need to develop new dyes of natural origin, especially in the food and cosmetic industries. Compared to dyes obtained synthetically, they may have a lower negative impact on human health and the environment [80]. The obtained extracts were used in the formulation of a model micellar make-up remover liquid. In each formulation, they were used in a concentration of 1%. The results of the color parameters of model cosmetics are presented in Table 6.

It was observed that the addition of 1% water extracts from PRE, PGE, CTE, and GGE significantly influenced the color of make-up remover. Each sample was clearly visible to the naked eye in color. The PRE extract changed the color of the cosmetic to orange, and the PGE, CTE, and GGE extracts changed it to yellow.

The possibility of using the analyzed extracts as potential dyes is confirmed by the relatively high values of ΔE_make-up remover with extract/base make-up remover_, which indicate a significant change in the color of model cosmetics with the addition of the extract compared to the base sample (without adding of the extracts). Literature data [73] show that if ΔE values are higher than 5, the color is perceived by the naked eye and perceived as a color effect. For make-up removers containing PRE, KTE, and CTE extracts, the ΔE values of 8.83, 9.17 and 8.14, respectively, were obtained. In the case of products with the extracts of PGE and GGE, no significant influence in the extract on the color of the preparation was observed. The values of ΔE are in the range 2.51–2.82. This means that the difference in color is only discernible to an experienced observer [80,81].

## 3. Materials and Methods

### 3.1. Plant Material and Extraction Procedure

The plant material used in the research was dry flowers of *P. rhoeas* L., *P. granatum* L., *C. ternatea* L., *C. tinctorius* L., and *G. globosa* L., which were obtained from the local herbal store. The extraction process was performed in an ultrasonic bath (Digital Mgtrasonic Cleaner, Berlin, Germany), which was carried out using the method described by Yang et al. [82]. 10 grams of dry flowers and 100 g of water were used to prepare water extracts of the tested plants. The process was carried out for 20 min at room temperature. The obtained extracts were then collected and filtered three times through Whatman No. 1 filter paper. After filtration, the extracts were evaporated under reduced pressure at 40 °C. A stock solution at the concentration of 100 mg/mL was prepared from the dried extracts and was stored in the dark at 4 °C until further analysis. The following abbreviations are used: PRE—*Papaver rhoeas* extract, PGE—*Punica granatum* extract, GGE—*Gomphrena globosa* extract, CTE—*Carthamus tinctorius* extract, KTE—*Clitoria ternatea* extract.

### 3.2. Determination of Bioactive Compounds by HPLC–UV-ESI–MS

The obtained extracts were analyzed to determine their main bioactive compounds using a HPLC (DionexUltiMate 3000 RS Thermo Fisher Scientific, Sunnyvale, CA, USA), coupled to mass spectrometer (4000 QTRAP, AB Sciex, Concord, ON, Canada), equipped with an electrospray ionization source (ESI) and a triple quadrupole-ion trap mass analyzer. Chromatographic separation was achieved with a gradient reverse-phase system. Furthermore, 100 × 4.6 mm chromatographic column Kinetex 3.5 µm XB-C18 100 Å with iso-butyl side chains and with TMS endcapping stationary phase used with similar composition guard column was purchased from Phenomenex and maintained at 30 °C. A binary solvent system comprising 0.1% (*v*/*v*) aqueous formic acid as solvent A and methanol as solvent B was used under gradient mode during 19.1 min of the run time. The elution conditions applied were as follow: 0.0–15.0 min 25–100% B, 15.0–17.0 min 100% B, 17.0–17.1 min 100–25% B, 17.1–19.1 min 25% B. The flow rate of the mobile phase was 0.6 mL/min and injection volume 10 μL. The eluent was monitored by electrospray ion mass spectrometer (ESI-MS) under negative ion mode and scanned from *m/z* 20 to 1000 Da. For quantification analysis, the triple quadrupole MS detector was working in multiple reaction monitoring (MRM) scan mode. Optimal mass analyzer conditions and the selection of product ions for individual compounds were determined experimentally. For this purpose, standard solutions of investigated compounds (1 ng/mL) in mobile phase composition were introduced using infusion pump operating in constant sample delivery. After ensuring that the correct precursor ion was selected, declustering potential (DP), entrance potential (EP), collision cell exit potential (CXP), and collision energy (CE) were optimized for each MRM transitions (Table S1). Two MRM transitions were monitored, one for quantification and one for confirmation. The MS parameters were set as follows: capillary temperature of 600 °C, curtain gas at 35 psi, nebulizer gas at 60 psi, and drying gas at 50 psi. Negative ionization mode source voltage −4500 V was applied for the determination of bioactive compounds. Nitrogen was used as curtain and collision gas. Data analysis was processed with Analyst 1.5.1 software. The identification of selected compounds was done by molecular mass and fragment of anion entries of each individual compound and confirmed by MS^2^ fragmentation. The identities of nine compounds were determined along with their chemical formula, deprotonated molecular ions, and the characteristic fragment ions for each individual peak. Six compounds were quantified based on the calibration curve generated using peak areas of the most intense MRM transitions of analytical standards. The linearity of the detector response for quantified compounds was demonstrated by injection of calibration standards at eight concentration levels ranging from 0.01 μg/mL to 2 μg/mL. Calibration curves were linear with the coefficients of correlation (R) greater than 0.99. In case the samples did not fall in the linear range of the MS detector, the samples were diluted.

Analytical standards of quinic acid, gallic acid, caffeic acid, caffeoylquinic acids (CQA, two isomers: 3- and 5-CQA), and quercetin were purchased from Sigma-Aldrich, St. Louis, MO, USA). All standards used were of analytical grade (≥99% purity).

Standard stock solutions were prepared by accurately weighing and dissolving 20 mg of each standard in 10 mL LC-MS grade methanol to give a concentration of 2 mg/mL. Serial dilutions of 2.0 μg/mL, 1.5 μg/mL, 1.0 μg/mL, 0.5 μg/mL, 0.1 μg/mL, 0.05 μg/mL, 0.02 μg/mL, and 0.01 μg/mL were then made using LC-MS grade methanol solution. Limit of quantitation (LOQ) was defined as 0.01 µg/ml.

LC-MS/MS assay was performed in triplicate. Obtained data were presented as means ± standard deviations.

### 3.3. Determination of Antioxidant Properties

#### 3.3.1. ABTS·+ Scavenging Assay

First, the ABTS solution was prepared by mixing 19.5 mg ABTS and 3.3 mg potassium persulfate with 7mL phosphate buffer (pH = 7.4) and dissolved for 16 h in darkness. Then, the solution was diluted to the absorbance at the level of about 1.0. Absorbances were measured at wavelength *λ* = 734 nm. Next, 20 mL of KTE, PGE, PRE, CTE, and GGE extracts (10, 100, 250, 500 µg/mL) was mixed with 980 mL of diluted ABTS•+ solution and then incubated for 10 min in darkness. In the next step, the absorbance of the prepared samples was measured at *λ* = 734 nm using a UV/VIS spectrophotometer Aquamate Helion (Thermo Fisher Scientific, Waltham, MA, USA). Distilled water was used as a blank. The ABTS·+ scavenging was calculated from Equation (1):

(1) $$\%\;\mathrm{of}\;\mathrm{ABTS}•+\mathrm{scavenging}=1-\frac{As}{Ac}\times 100$$

where: As—absorbance of the sample; Ac—absorbance of the control sample. Measurements were carried out in triplicate for each extract sample. The procedure was described by Gaweł-Beben et al. [83].

#### 3.3.2. DPPH Radical Scavenging Assay

The ability of the extracts to scavenge free radicals was carried out using the method described by Brand-Williams et al. [84]. It is based on the use of the 1,1-diphenyl-2-picrylhydrazyl (DPPH) radical. First, 33 µL of aqueous solutions of extracts at concentrations of 100 µg/mL were mixed with 167 µL methanol solution of DPPH (4 mM) and transferred to a 96-well plate, then mixed by shaking. Afterwards, the absorbance of the samples was measured at wavelength of 517 nm. Measurements were made every 5 min for 30 min on a UV-VIS Filter Max *λ* = 5 spectrophotometer (Thermo Fisher Scientific, Waltham, MA, USA). Three independent replicates were performed for each extract. Water with a DPPH solution was used as a control. The antioxidant capacity was expressed as a percentage of DPPH inhibition using Equation (2):

(2) $$\%\mathrm{DPPH}\;\mathrm{scavenging}=\frac{\mathrm{Abs}\;\mathrm{control}-\mathrm{Abs}\;\mathrm{sample}}{\mathrm{Abs}\;\mathrm{control}}\times 100$$

where: As—absorbance of the sample; Ac—absorbance of the control sample. Measurements were carried out in triplicate for each extract sample.

#### 3.3.3. Detection of Intracellular Levels of Reactive Oxygen Species (ROS)

To determine the ability of the analyzed extracts to generate the intracellular production of reactive oxygen species in HaCaT and BJ cells, a fluorogenic H_2_DCFDA dye was used. This compound has the ability to enter cells by passive diffusion, where it is deacetylated by intracellular esterases to a non-fluorescent compound. If reactive oxygen species are present in the cell, this compound is transformed into highly fluorescent DCF. To determine the intracellular level of ROS in HaCaTs and BJ, cells were seeded in 96-well plates. Then, cells were cultured in an incubator for 24 h. DMEM medium was removed and replaced with 10 µM H_2_DCFDA (Sigma Aldrich, St. Louis, MO, USA) dissolved in serum free DMEM medium. HaCaT and BJ cells were incubated in H_2_DCFDA for 45 min and then incubated with the extracts in the concentrations: 100, 250, and 500 µg/mL. Cells treated with 1 mM hydrogen peroxide (H_2_O_2_) were used as positive controls. The control samples were cells untreated with the tested extracts. DCF fluorescence was measured every after 90 min using a FilterMax F5 microplate reader (Thermo Fisher Scientific) at a maximum excitation of 485 nm and emission spectra of 530 nm [85].

### 3.4. Assessment of Matrix Metallopeptidases Inhibition

#### 3.4.1. Determination of Anti-Elastase Activity

To determine the possibility of inhibiting matrix metalloproteinase, neutrophil elastase (NE), a fluorometric kit (Abcam, ab118971) was applied. The test was carried out in accordance with the instructions attached to the kit and with the procedure described by Nizioł-Łukaszewska et al. [86]. Analyses were performed in a standard 96-well plate with a clear flat bottom. For the analysis, plant extracts in a concentration of 100 and 250 µg/mL were used. Initially, NE enzyme solutions, an NE substrate, and an inhibitor control (SPCK) were prepared according to the instructions. Diluted NE solution was added to all wells and then, test samples, the inhibitor control, and the enzyme control (Assay Buffer) were added to subsequent wells. Afterwards, samples were mixed and incubated at 37 °C for 5 min. In the meantime, a reaction mixture was prepared by mixing the Assay Buffer and NE substrate. The mixture was added to each well and mixed thoroughly. Fluorescence was measured immediately at excitation wavelength *λ* = 400 nm and emission *λ* = 505 nm using a microplate reader (FilterMax F5, Thermo Fisher Scientific, Waltham, MA, USA). The ability to inhibit NE activity of the analyzed samples was calculated from Equation (3):

(3) $$\%\mathrm{relative}\;\mathrm{NE}\;\mathrm{activity}=\frac{\Delta \mathrm{RFU}\;\mathrm{test}\;\mathrm{inhibitor}}{\Delta \mathrm{RFU}\;\mathrm{enzyme}\;\mathrm{control}}\times 100$$

The final result was the arithmetic mean of three independent measurements.

#### 3.4.2. Determination of Anti-Collagenase Activity

To assess the ability of the obtained extracts to inhibit collagenase activity, a fluorometric kit (Abcam, Cambridge, UK, ab211108) was applied. The test was carried out in accordance with the instructions attached to the kit and with the procedure described by Nizioł-Łukaszewska et al. [86]. Analyses were performed in a standard 96-well plate with a clear flat bottom. For the analysis, plant extracts in a concentration of 100 and 250 µg/mL were used. First, collagenase (COL) was dissolved in a collagenase analysis buffer (CAB). Then, analyzed samples were added to COL and CAB. Inhibitor control samples were prepared by mixing the collagenase inhibitor (1,10-phenanthroline (80 mM)) with collagenase and CAB buffer. Enzyme control wells were prepared by mixing diluted COL with CAB. The CAB buffer was used as a background control. Then, samples were incubated at room temperature for 15 min. A reaction mixture was prepared by mixing the collagenase substrate with CAB. The reaction mixture prepared in this way was added to all analyzed samples and mixed thoroughly. Afterwards, fluorescence was measured at excitation wavelength 490 nm and emission 520 nm. The measurement was performed in kinetic mode for 60 min at 37 °C. The ability to inhibit COL activity of obtained extracts was calculated by Equation (4):

(4) $$\%\mathrm{relative}\;\mathrm{COL}\;\mathrm{inhibition}=\frac{\mathrm{enzyme}\;\mathrm{control}-\mathrm{sample}}{\mathrm{enzyme}\;\mathrm{control}}\times 100$$

### 3.5. Determination of Anti-Inflammatory Properties

#### 3.5.1. Inhibition of Protein Denaturation

Proteinase inhibitory activity of PRE, PGE, KTE, CTE, and GGE extracts was performed according to the method of Sakat et al. [87], which was modified by Gunathilake et al. [88]. Briefly, the reaction solution (2 mL) consisted of 1 mL of 1% trypsin in 20 mM Tris-HCl buffer (pH 7.4) and 1 mL of test sample (0.02 mL extract 0.980 mL water). The solution was incubated (37 °C for 5 min), and then 1 mL of 0.8% (*w*/*v*) casein was added and the mixture was further incubated for 20 min. At the end of the incubation, 2 mL of 70% perchloric acid was added to complete the reaction. The mixture was centrifuged, and the absorbance of the supernatant was measured at 210 nm against the buffer as a blank. Phosphate buffer solution was used as control. The percentage inhibition of protein denaturation was calculated using the following formula:

(5) $$\%\mathrm{inhibition}\;\mathrm{of}\;\mathrm{proteinase}\;\mathrm{activity}=100\times \left(1-\frac{A2}{A1}\right)$$

where A1 = absorption of the control sample, and A2 = absorption of the test sample.

#### 3.5.2. Inhibition of Lipoxygenase Activity

The ability of obtained extracts to inhibit lipoxygenase activity was determined using the method described by Sarvesvaran et al. [89]. First, 10 µL of plant extracts in different concentrations (100, 250 and 500 µg/mL) were mixed in 96-well plate with 160 µL of 100 mM PBS and 20 μL of soybean lipoxygenase solution (167 U/mL). Samples were incubated at 25 °C for 10 min and after this time 10 µL of sodium linoleic acid was added to initiate the reaction. Then, absorbance of samples was measured at 234 nm over a period of 3 min in every minute using a FilterMax F5microplate reader (Thermo Fisher Scientific, Waltham, MA, USA). Diclophenac was used as positive control. The percent of lipoxygenase activity inhibition was calculated from Equation (6):

(6) $$\%\mathrm{inhibition}\;\mathrm{of}\;\mathrm{lipoxygenase}\;\mathrm{activity}=\frac{Ac-As}{Ac}\times 100$$

where: As is the absorbance of the tested sample, Ac is the absorbance of negative control.

The final result was the arithmetic mean of three independent measurements.

### 3.6. Cytotoxicity Analysis

#### 3.6.1. Cell Culture

In this study, two skin cell lines were used: normal human keratinocytes (HaCaT) and fibroblasts (BJ). HaCaTs were obtained from CLS Cell Lines Service (CLS Cell Lines Service GmbH, Eppelheim, Germany) and BJs from the American Type Culture Collection (Manassas, VA, USA). Cells were grown in Dulbecco’s Modification of Eagle’s Medium (DMEM, Biological Industries, Cromwell, CO, USA) with sodium pyruvate, L-glutamine, and high glucose content (4.5 g/L). Medium was also enriched with 10% fetal bovine serum (Gibco, Waltham, MA, USA) and 1% with antibiotics (100 U/mL penicillin and 1000 µg/mL streptomycin, Gibco) to prevent microbial contamination. Cells were grown in an incubator at 37 °C in a humidified atmosphere of 95% air and 5% carbon dioxide.

#### 3.6.2. Alamar Blue Assay

After the cultured cells (HaCaT and BJ) had reached the desired confluence, the DMEM medium was aspirated in the culture flasks. The bottom-attached cells were washed twice with sterile phosphate buffered saline. The cell layer was detached with trypsin and then the cells were placed in fresh DMEM medium. Cells were plated in 96-well flat bottom plates (VWR, Radnor, PE, USA) and after attaching to the bottom of the plates, cells were treated with extracts (100, 250 and 500 µg/mL). Cells were incubated for 24 h. 

The cytotoxicity test was performed with the Alamar Blue assay (Sigma, R7017, Life Technologies, Bleiswijk, The Netherlands). After incubation, a resazurin solution at a concentration of 60 µM was added to the wells, then plates were placed in an incubator at 37 °C for 2 h. After this time, fluorescence has been measured (*λ* = 570 nm). Each extract concentration was performed in three replications.

#### 3.6.3. Neutral Red Uptake Assay

Neutral Red Uptake Assay is the second test used to determinate the cytotoxicity of PRE, PGE, KTE, CTE, and GGE. First, 96-well flat bottom plates were prepared as described in the previous section. After 24 h of exposure of the cells to the extracts, they were aspirated and replaced with neutral red dye (40 µg/mL) and incubated for 2 h. After this time, cells were washed with phosphate buffered saline. In the next step, decolorizing buffer (150 µL) was added to wells. Then, the uptake of neutral red dye was determined by measuring the optical density (OD) at 540 nm. Each extract concentration was performed in three replications.

### 3.7. Determination of Sun Protection Factor (In Vitro)

The sun protection factor (SPF) was determined by measuring the absorbance of aqueous solution of extracts in concentrations of 10 µg/mL and 50 µg/mL, within the wavelength range from 290 to 320 nm at 5-nm intervals. From the obtained results, the SPF was calculated from the Mansur Equation [90]:

(7) $$\mathrm{SPF}=\mathrm{CF}\times \overset{320}{\underset{290}{\sum }}\left[EE\left(\lambda \right)\times I\left(\lambda \right)\times (\mathrm{ABS}\left(\lambda \right)\right]$$

where: EE(*λ*)—erythemal effect spectrum, I(*λ*)—solar intensity spectrum, ABS(*λ*)—absorbance of sunscreen product, CF—correction factor (=10), E(*λ*) × I(*λ*)—values determined by Sayre were used [91].

### 3.8. Transepidermal Water Loss (TEWL) and Skin Hydration Measurements

TEWL and skin hydration measurements were conducted using a TEWAmeter TM 300 probe and Corneometer CM 825 probe connected to an MPA adapter (Courage + Khazaka Electronic, Köln, Germany). Five volunteers participated in the study. On their forearm skin, six areas (2 × 2 cm in size) were marked. An amount of 0.2 mL of the tested plant extracts was applied in five places, the sixth place was the control (not treated with any samples). After 60 and 360 min, the hydration level and TEWL measurements were taken. The final result was the arithmetic mean (from each volunteer) of five independent measurements (skin hydration) and 20 measurements (TEWL).

### 3.9. Preparation of Model Cosmetics (Make-Up Remover) Containing Extracts

A model cosmetic (make-up remover) was prepared. All the components used were in line with EcoCert and COSMOS requirements. The formulation is shown in Table 7.

The product was produced by mixing the ingredients (from item 1 to item 6) at room temperature until a homogeneous liquid was obtained. In the last step, the pH of the formulation was adjusted. The make-up remover was divided into portions. An amount of 1% of the stock solutions of the extract was added to each portion and mixed thoroughly.

### 3.10. Determination of the Color Parameters of Extracts and Cosmetics (Make-Up Removers) Containing Extracts

Samples of extracts and cosmetics with extracts were tested at room temperature, 48 h after their preparation. A CHROMA METER CR-400 (Konica Minolta, Sensing Inc., Tokyo, Japan) was used to evaluate the color parameters (CIELAB coordinates). The CIELAB system was defined by the International Commission on Illumination in 1978. It is based on three color attributes: L*, a*, b*, where L* is a brightness variable proportional to the value in the Munsell system, and a* and b* are chromatic coordinates. The a* and b* coordinates indicate positions on the red/green and yellow/blue axes, respectively (+a = red, −a = green; + b = yellow, −b = blue).

Based on the data obtained: L*, a* and b*, the following color parameters were calculated: chroma (C*) and hue (*h**^o^***). The following equations were used:

(8) $$C^{*}=\sqrt{{\left(a^{*}\right)}^{2}+{\left(b^{*}\right)}^{2}}$$

(9) $$h^{o}=arctan\frac{b^{*}}{a^{*}}$$

General color difference (ΔE_make-up remover with extract+extract/base make-up remover_) was calculated according to the following formula:

(10) $${\Delta E}_{\mathrm{make}-\mathrm{up}\;\mathrm{remover}\;\mathrm{with}\;\mathrm{extract}\;/\;\mathrm{base}\;\mathrm{make}-\mathrm{up}\;\mathrm{remover}}^{*}=\sqrt{{\left({\Delta L}^{*}\right)}^{2}+{\left({\Delta a}^{*}\right)}^{2}+{\left({\Delta b}^{*}\right)}^{2}}$$

where: ΔL*, Δa*, and Δb* are the mathematical differences between make-up remover with extracts L*, a*, b* and base make-up remover L*, a*, b* values.

## 4. Conclusions

Based on the obtained results, it can be concluded that the tested plant extracts show several positive features, thanks to which they can be used in the production of cosmetics as their safe and bioactive ingredient. It has been shown that these plants are a rich source of polyphenols, which gives them antioxidant properties. PRE showed the best ability to scavenge free radicals, which is probably because it contains the most polyphenols compared to other plants. PGE and PRE showed the best ability to reduce ROS production in cells. Moreover, plants do not show any cytotoxic activity. All tested extracts showed an inhibitory effect on the elastase and collagenase enzymes, with *P. granatum* and GGE having the greatest inhibitory effect. This may indicate that these plants can be used in cosmetics as substances that slow down the aging processes. Moreover, the obtained results indicate that these plants have anti-inflammatory properties. In this case, CTE and KTE appeared to be the best. KTE and PGE showed a protective effect against UV radiation, even at low concentrations. At higher concentrations, all plants had a UV protective effect. Furthermore, the plants had a positive effect on skin hydration and reduce transepidermal water loss. PRE, KTE, and CTE extracts may be used as effective colorants in cosmetics products. The model liquid for make-up removal containing above mentioned extracts were characterized by an intense and stable color over time. Color of the cosmetics with PGE and GGE extracts can be noticed only by experienced observers, and they are not significantly differing from the blank cosmetic sample, without the addition of the extract. Considering all the obtained results, it can be concluded that *Papaver rhoeas*, *Clitoria ternatea*, and *Carthamus tinctorius* can be successfully used as sources of yellow, orange, blue, and purple dyes in the production of cosmetics that will be safe to use, and, what is more, will have a positive effect on the skin.

## Figures and Tables

**Table 1:** Polyphenols detected using HPLC-ESI-MS in PRE, PGE, KTE, CTE, and GGE extracts in water.

Retention Time (min)	Molecular Formula	Molar Mass (Da)	Precursor Ion *m*/*z*	Main Product Ions MS^2^ *m*/*z*	Identification	PRE	PGE	KTE	CTE	GGE
1.6	C_7_H_12_O_6_	192.2	191 [M − H]^−^	127 [M − H − H_2_O − HCOOH]^−^, 93 [M − C_4_H_3_O_3_]^−^, 85 [M − C_3_H_7_O_4_]^−^, 59 [M − C_5_H_9_O_4_]^−^	Quinic acid	x	x	x	x	x
2.1	C_7_H_6_O_5_	170.1	169 [M − H]^−^	151 [M − H − H_2_O]^−^, 125 [M − H − CO_2_]^−^, 107 [M − H − CO_2_ − H_2_O]^−^, 79 [M − C_6_H_3_O]^−^	Gallic acid	x	x	x	x	x
2.8	C_16_H_18_O_9_	354.3	353 [M − H]^−^	191 [M − 3H_2_O − C_6_H_5_O_2_]^−^, 179 [M − 3H_2_O − C_6_H_4_ − COOH]^−^, 85 [M − C_15_H_12_O_2_ − COOH]^−^	5-Caffeoylquinic acid	-	-	x	x	-
4.2	C_16_H_18_O_9_	354.3	353 [M − H]^−^	191 [M − 3H_2_O − C_6_H_5_O_2_]^−^, 179 [M − 3H_2_O − C_6_H_4_ − COOH]^−^, 85 [M − C_15_H_12_O_2_ − COOH]^−^	3-Caffeoylquinic acid	-	x	x	x	x
5.1	C_9_H_8_O_4_	180.2	179 [M − H]^−^	135 [M − COOH]^−^, 107 [M − C_3_H_5_O_2_]^−^	Caffeic acid	x	x	x	x	x
7.1/8.1	C_27_H_30_O_16_	610.5	609 [M − H]^−^	300 [M − H − C_12_H_21_O_9_]^−^	Rutin	x	x	x	x	x
8.5	C_27_H_30_O_15_	594.5	593 [M − H]^−^	383 [M − C_8_H_19_O_6_]^−^, 352 [M − C_9_H_21_O_7_]^−^, 284 [M − C_12_H_22_O_9_]^−^	Kaempferol-3-*O*-rutinoside	x	x	x	x	x
10.0	C_15_H_10_O_7_	302.2	301 [M − H]^−^	179 [M − H − C_7_H_6_O_2_]^−^, 151 [M − C_8_H_7_O_3_]^−^, 121 [M − C_8_H_5_O_5_]^−^, 107 [M − C_9_H_5_O_5_]^−^	Quercetin	x	x	x	x	x
11.3	C_21_H_20_O_11_	448.3	447 [M − H]^−^	284 [M − H_2_O − C_6_H_10_O_4_]^−^, 255 [M − C_6_H_9_O_7_]^−^, 179 [M − C_15_H_9_O_5_]^−^	Kaempferol-3-*O*-glucoside	x	x	x	x	x

**Table 2:** HPLC-ESI-MS/MS quantitative analysis of PRE, PGE, KTE, CTE, and GGE water extracts. Values are means ± SD of triplicate.

Compound	Content [µg/mL]				
	PRE	PGE	KTE	CTE	GGE
Quinic acid	0.11 ± 0.01	0.78 ± 0.03	1.85 ± 0.01	7.71 ± 0.01	0.05 ± 0.00
Gallic acid	0.20 ± 0.02	22.00 ± 0.28	1.15 ± 0.01	0.57 ± 0.00	0.12 ± 0.01
Caffeic acid	0.31 ± 0.02	0.13 ± 0.00	4.96 ± 0.00	1.22 ± 0.02	0.39 ± 0.01
5-CQA	-	-	0.02 ± 0.00	0.03 ± 0.00	0.01 ± 0.00
3-CQA	-	1.75 ± 0.02	0.77 ± 0.04	0.37 ± 0.02	0.08 ± 0.00
Quercetin	0.14 ± 0.00	<LOQ	3.45 ± 0.13	0.08 ± 0.00	0.93 ± 0.04
**Sum of quantified compounds**	**0.76**	**24.66**	**12.2**	**9.98**	**1.58**

LOQ—limit of quantitation (0.01 µg/mL).

**Table 3:** Values of IC_50_ of ABTS for PRE, PGE, GGE, CTE, and KTE extracts.

Sample	PRE	PGE	KTE	CTE	GGE
IC_50_ (µg/mL)	65.5 ± 1.42	24.8 ± 0.42	63.3 ± 1.03	100.5 ± 3.22	134.3 ± 4.18

**Table 4:** Values of IC_50_ of DPPH for PRE, PGE, GGE, CTE, and KTE extracts.

Sample	PRE	PGE	KTE	CTE	GGE
IC_50_ (µg/mL)	88.5 ± 2.59	47.5 ± 1.01	87.2 ± 3.46	150.6 ± 5.61	205.2 ± 4.22

**Table 5:** Color parameters for water extracts (concentration of 100 mg/mL): PRE, PGE, KTE, CTE, GGE. Values are means of three replicate determinations (*n* = 3) ± SD.

	L*	a*	b*	C*	*h^o^*	
**PRE**	23.23 ± 0.06	2.51 ± 0.10	−1.18 ± 0.01	2.8 ± 0.02	−25.2 ± 0.05	red-purple
**PGE**	27.81 ± 0.15	1.29 ± 0.01	0.26 ± 0.03	1.3 ± 0.03	11.4 ± 0.06	weak color, reddish hue
**KTE**	22.86 ± 0.19	1.96 ± 0.01	−1.93 ± 0.02	2.8 ± 0.04	315.5 ± 0.09	blue–violet
**CTE**	26.91 ± 0.11	1.41 ± 0.02	5.00 ± 0.02	5.2 ± 0.10	74.3 ± 0.13	yellow
**GGE**	28.8 ± 0.08	1.10 ± 0.02	0.69 ± 0.03	1.3 ± 0.08	32.1 ± 0.12	weak color, slightly orange hue

**Table 6:** Color parameters for cosmetic (make-up remover) with water extracts: PRE, PGE, KTE, CTE, GGE. Values are means of three replicate determinations (*n* = 3) ± SD.

	L*	a*	b*	C*	*h^o^*	ΔE Cosmetic + Extract/Base Cosmetic	
**Cosmetic with PRE**	16.35 ± 0.04	3.88 ± 0.05	3.34 ± 0.03	5.1 ± 0.04	40.7 ± 0.11	8.83 ± 0.03	orange
**Cosmetic with PGE**	22.64 ± 0.11	−0.31 ± 0.04	3.04 ± 0.01	3.1 ± 0.03	95.8 ± 0.06	2.51 ± 0.03	yellow
**Cosmetic with KTE**	14.48 ± 0.09	0.56 ± 0.03	−0.70 ± 0.01	0.9 ± 0.03	308.8 ± 0.05	9.17 ± 0.04	weak color, blue–violet
**Cosmetic with CTE**	15.84 ± 0.18	0.26 ± 0.04	3.49 ± 0.02	3.5 ± 0.02	94.2 ± 0.03	8.14 ± 0.04	yellow
**Cosmetic with GGE**	21.64 ± 0.19	0.73 ± 0.04	2.83 ± 0.02	2.9 ± 0.03	104.5 ± 0.05	2.82 ± 0.03	yellow
**Base cosmetic**	23.47 ± 0.17	−0.63 ± 0.04	0.69 ± 0.02	0.9 ± 0.06	132.4 ± 0.05	-	weak color, yellow-green

**Table 7:** Formulation of a model cosmetic (make-up remover).

	Ingredient (INCI Name)	Raw Material (Trade Name, Supplier)	(wt.%)
**1**	Polyglyceryl-4 Laurate/Sebacate, Polyglyceryl-6 Caprylate/Caprate, Water	Natragem S140 (Croda)	6.0
**2**	Caprylyl/Capryl Glucoside	Plantacare 810 (BASF)	6.0
**3**	Glycerin	Glycerin VegeTable 99.7 (local supplier)	1.0
**4**	Propanediol	Propanediol natural (Cosphaderm)	3.0
**5**	Aqua	Deionised water	to 100
**6**	Benzyl Alcohol, Benzoic Acid, Dehydroacetic Acid, Tocopherol	Euxyl K903 (Schülke & Mayr)	0.5
**7**	Lactic Acid	Lactic Acid (local supplier)	to pH 5.5

## Data Availability

Data is contained within the article.

## Supplementary Materials

Supplementary Materials (chromatograms) are available online.

## Sample Availability

Samples of the compounds used, and the plants extracts tested are available from the authors.
