# Supplementary material for: Flower Extracts as Multifunctional Dyes in the Cosmetics Industry
Source: Molecules. 2022 Jan 29;27(3):922. doi: 10.3390/molecules27030922 (PMC8838747; doi:10.3390/molecules27030922)
Supplement: Supplementary file 1 [file molecules-27-00922-s001.zip › molecules-1553193-supplementary.pdf]

Article

Flower extracts as multifunctional dyes in the cosmetics industry

Supplementary material

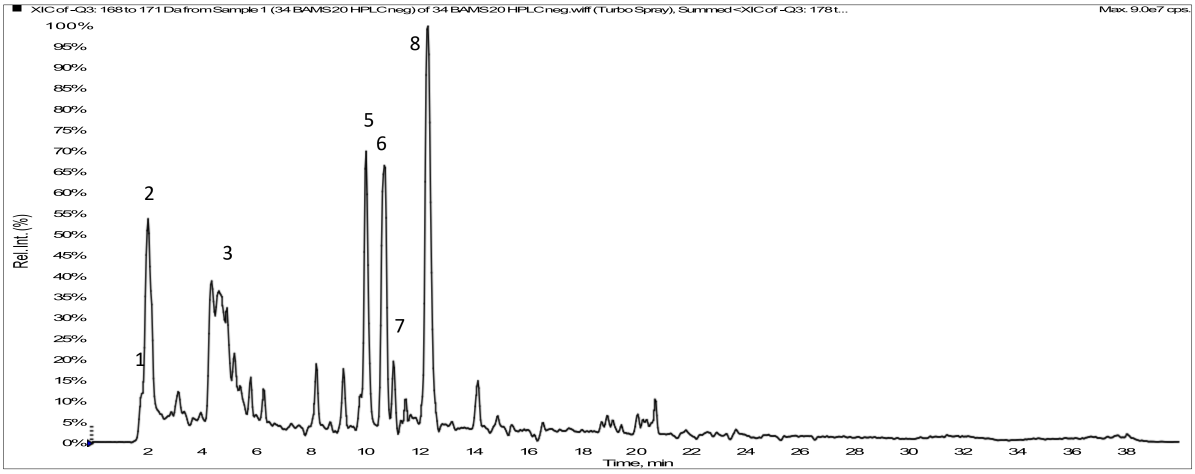

Figure S1**.** Extracted ion chromatograms (XIC) obtained for PRE.

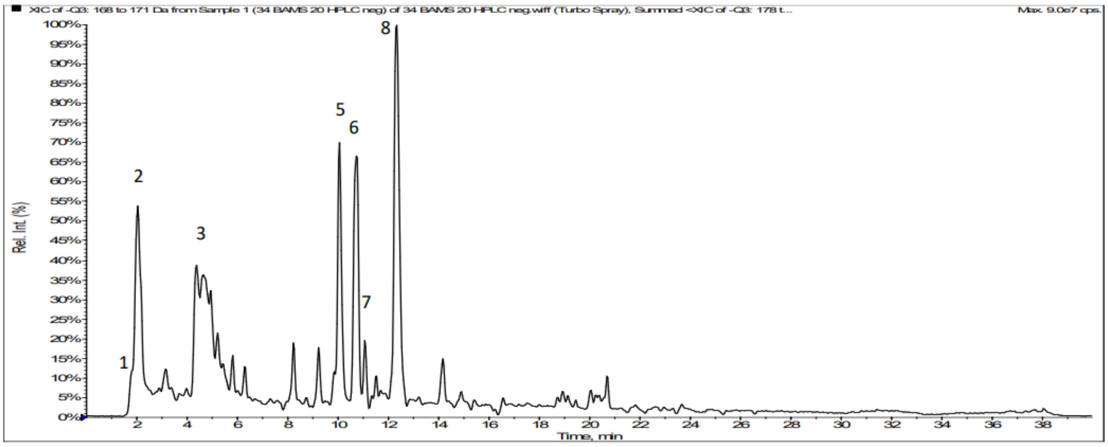

Figure S2**.** Extracted ion chromatograms (XIC) obtained for KTE.

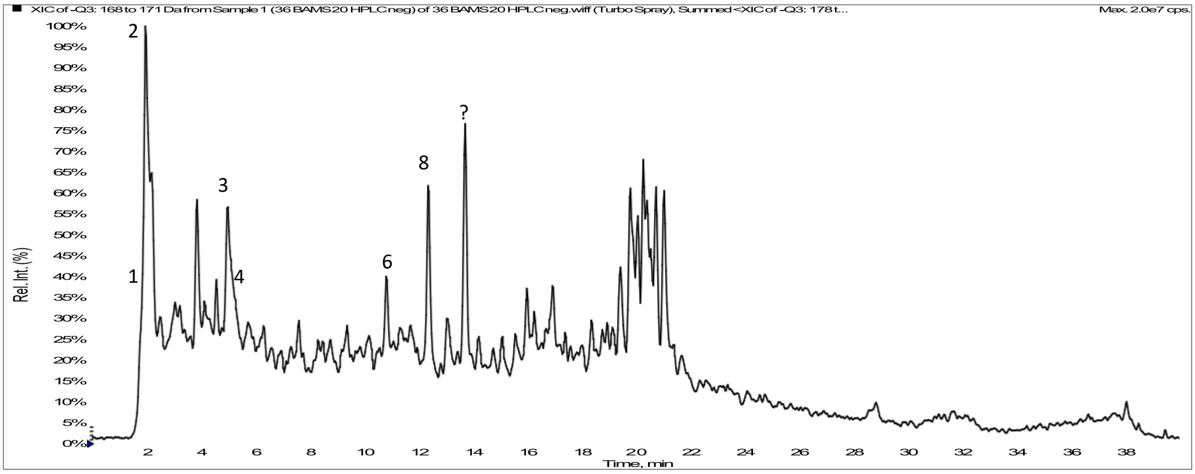

Figure S3**.** Extracted ion chromatograms (XIC) obtained for GGE.

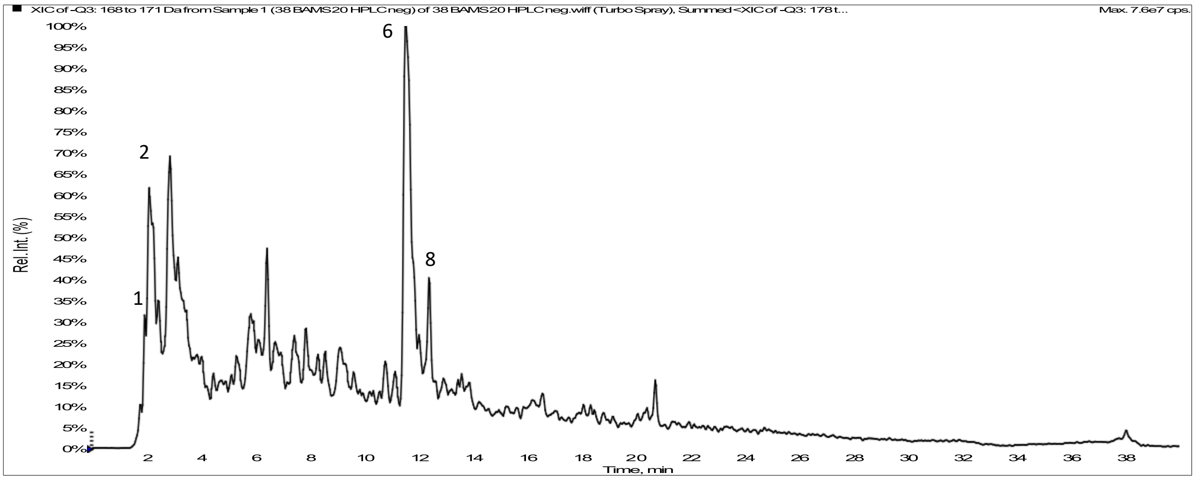

Figure S4**.** Extracted ion chromatograms (XIC) obtained for PGE.

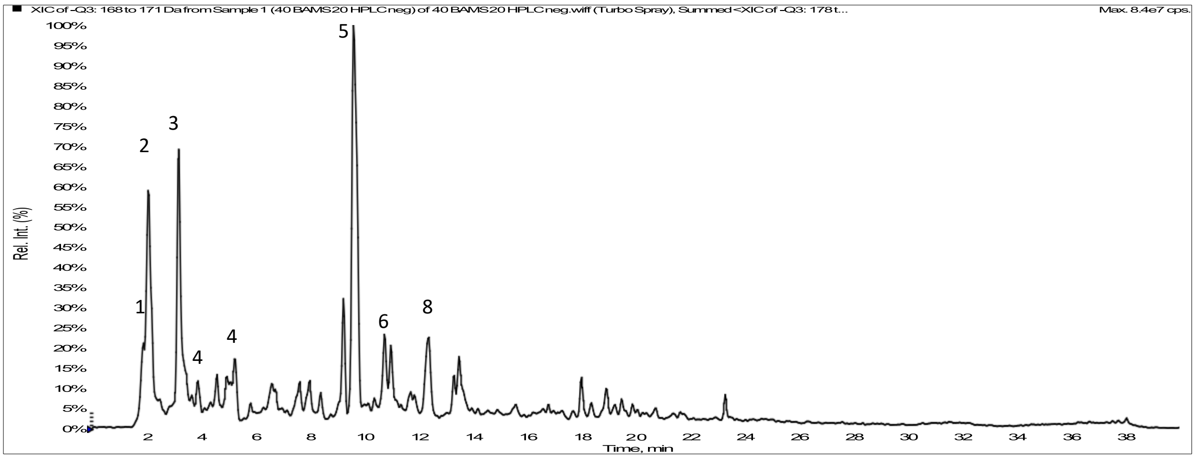

Figure S5**.** Extracted ion chromatograms (XIC) obtained for CTE.

Table S1. Polyphenols detected using HPLC-ESI-MS

No.	Retention time (min)	Molar mass (Da)	Precursor ion m/z	Identification
1	1.9	180.2	179 $[M-H]^-$	Caffeic acid
2	2.0	192.2	191 $[M-H]^-$	Quinic acid
3	5	170.1	169 $[M-H]^-$	Gallic acid
4	4.0<br>5.2<br>5.8	354.3	353 $[M-H]^-$	Caffeoylquinic acids
5	10.7	464.1	463	Isoquercetin
6	10.8	302.2	301 $[M-H]^-$	Quercetin
7	10.7	610.5	609 $[M-H]^-$	Rutin
8	12.5	448.3	447 $[M-H]^-$	Kaempferol-O-glucoside
